# Case Study of an Outbreak: Resident, Staff, and Community Indicators

**DOI:** 10.1093/geroni/igab046.226

**Published:** 2021-12-17

**Authors:** Toni Miles

**Affiliations:** College of Public Health, University of Georgia, Georgia, United States

## Abstract

Outbreak investigation is not infection control. We present a self-study of factors influencing outcomes inside a single nursing home during the early stage of the outbreak - February to May 2020. We examine 3 sources of influence: Practice / Operations; Local, State & Federal Policies; Uncontrollable operational factors. Outcomes of interest include: mortality and resident / staff health. Data consists of clinical records, review of communications, and interviews with staff present during the critical period. Infection control is different from outbreak investigation. There must be a balance between staff empowerment and adherence to guidelines. In an outbreak, staff need the confidence to make decisions based on incomplete knowledge. The presentation concludes with lessons learned – what worked and what actions need improvement. There are areas requiring further analyses of policy and ethics.

